# Metabolomics profiling in plasma samples from glioma patients correlates with tumor phenotypes

**DOI:** 10.18632/oncotarget.7974

**Published:** 2016-03-07

**Authors:** Hua Zhao, Amy B. Heimberger, Zhimin Lu, Xifeng Wu, Tiffany R. Hodges, Renduo Song, Jie Shen

**Affiliations:** ^1^ Department of Epidemiology, The University of Texas MD Anderson Cancer Center, Houston, TX 77030, USA; ^2^ Division of Neuro-Surgery, The University of Texas MD Anderson Cancer Center, Houston, TX 77030, USA; ^3^ Department of Neuro-Oncology, The University of Texas MD Anderson Cancer Center, Houston, TX 77030, USA

**Keywords:** metabolomics, glioma, tumor phenotype

## Abstract

**Background:**

Tumor-based molecular biomarkers have redefined in the classification gliomas. However, the association of systemic metabolomics with glioma phenotype has not been explored yet.

**Methods:**

In this study, we conducted two-step (discovery and validation) metabolomic profiling in plasma samples from 87 glioma patients. The metabolomics data were tested for correlation with glioma grade (high vs low), glioblastoma (GBM) versus malignant gliomas, and *IDH* mutation status.

**Results:**

Five metabolites, namely uracil, arginine, lactate, cystamine, and ornithine, significantly differed between high- and low-grade glioma patients in both the discovery and validation cohorts. When the discovery and validation cohorts were combined, we identified 29 significant metabolites with 18 remaining significant after adjusting for multiple comparisons. Those 18 significant metabolites separated high- from low-grade glioma patients with 91.1% accuracy. In the pathway analysis, a total of 18 significantly metabolic pathways were identified. Similarly, we identified 2 and 6 metabolites that significantly differed between GBM and non-GBM, and *IDH* mutation positive and negative patients after multiple comparison adjusting. Those 6 significant metabolites separated *IDH1* mutation positive from negative glioma patients with 94.4% accuracy. Three pathways were identified to be associated with *IDH* mutation status. Within arginine and proline metabolism, levels of intermediate metabolites in creatine pathway were all significantly lower in *IDH* mutation positive than in negative patients, suggesting an increased activity of creatine pathway in *IDH* mutation positive tumors.

**Conclusion:**

Our findings identified metabolites and metabolic pathways that differentiated tumor phenotypes. These may be useful as host biomarker candidates to further help glioma molecular classification.

## INTRODUCTION

During the past decade, glioma classification has moved from histopathology only to molecular characterization. Allelic loss of 1p/19q and *p53* gene mutation has been deemed as the hallmarks for low grade oligodendroglioma and astrocytomas, respectively [1, 2]. The results from The Cancer Genome Atlas (TCGA) Research Network and several other studies have pinpointed phosphoinositide 3-kinase (PI3K), RTK/RAS/PI3K, EGF receptors (EGFR), p53, retinoblastoma (RB), and PTEN signaling alterations as driving forces for high-grade glioma tumorigenesis [3, 4].

The renewed interest of Warburg effect has drawn attention to the understanding of how underlying metabolic alterations may contribute to the aggressive phenotype in tumors [5, 6]. Although the data are still limited, promise has already been shown of using metabolomics in characterizing gliomas [7]. For example, utilizing metabolomic profiling in 69 Grade II to IV glioma tumor tissues, Chinnaiyan et al. identified a metabolic classifier that could group glioma tumors into 3 different subclasses with distinct prognostic relevance [7]. Metabolomic platforms quantify small-molecule metabolites in biospecimens and can be used to evaluate the role of metabolic alterations in chronic disease. Because it takes into account genetic regulation, altered kinetic activity of enzymes, genomics and proteomics, metabolomics reflects changes in phenotype, and thereby function [8, 9]. Studies using metabolomics in various cancers have shown that there are common alterations in metabolism in patients with cancer, but there are also disease specific alterations in metabolism [10–14].

It has recently become clear that altered metabolic homeostasis plays important roles in carcinogenesis. Recent results from limited clinical and epidemiological studies have suggested that metabolic disorders may affect the progression of high grade gliomas. For example, Derr et al. reported that high grade gliomas patients with hyperglycemia have a shortened overall survival [15]. Chambless et al. observed that pre-existing diabetes and elevated body mass index (BMI) are independent risk factors for high grade glioma progression [16]. However, to our knowledge, there have been no studies to date examining the role of small-molecule metabolites in the circulation in relation to glioma characterization. In the current study, utilizing targeted metabolomics analysis, we analyzed 224 known metabolites from 25 key metabolic pathways in plasma samples from 87 glioma patients. We hypothesized that plasma metabolite profiles could differentiate glioma tumor phenotypes.

## RESULTS

Basic demographic characteristics of the patient cohort were shown in Table 1. Briefly, the mean age was 45 years old, and nearly 60% of the study subjects were male. The majority of study subjects were Caucasians (86.2%). About 20% of the study subjects used steroid during the treatment. Seizure medication use was prevalent (72.4%). In addition, 44.8% of the study subjects had dyslipidemia diagnosis.

**Table 1:** Demographic characteristics of the patient cohort

Age, mean (range)	45 (22–72)
**Gender (%)**	
Male	50 (57.5)
Female	37 (42.5)
**Ethnicity (%)**	
Caucasian	75 (86.2)
Others	10 (11.5)
Unknown	2 (2.3)
KPS, mean (range)	92 (60–100)
**Steroid use (%)**	
Yes	18 (20.7)
No	69 (79.3)
**Seizure Medication Use (%)**	
Yes	63 (72.4)
No	24 (27.6)
**Dyslipidemia during study (%)**	
Yes	22 (25.3)
No	4 (4.6)
Test not done	61 (70.1)
**Dyslipidemia diagnosis (%)**	
Yes	39 (44.8)
No	48 (55.2)
**Antiglycemic medication Use**	
Yes	5 (5.7)
No	82 (94.3)

**Figure 1:**
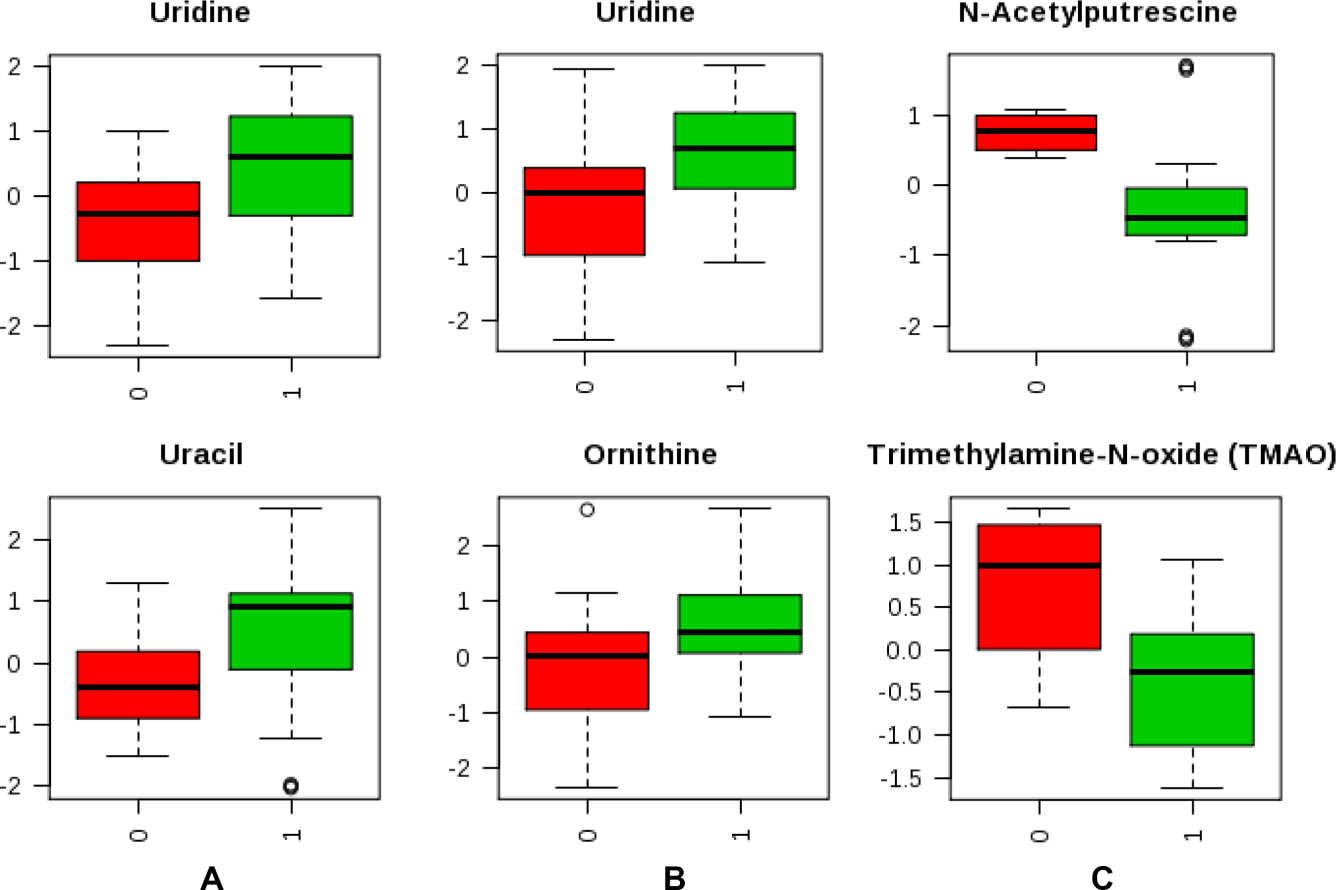
Box plot of top 2 metabolites that significantly differed between tumor characteristics (**A**) High and low grade (1 vs 0); (**B**) GBM and malignant gliomas (1 vs 0); (**C**) *IDH* mutation positive and negative status (1 vs 0).

**Figure 2:**
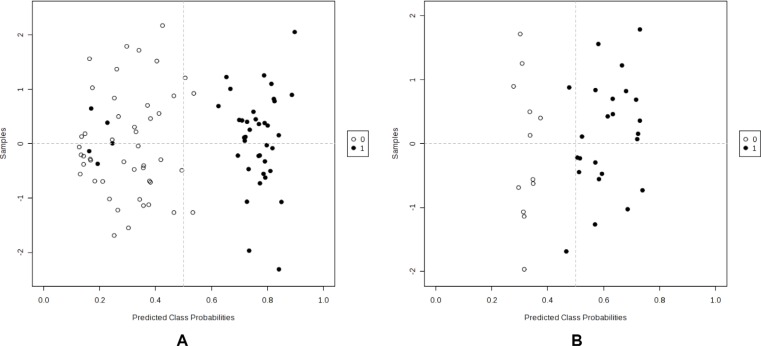
PLS-DA analysis to differentiate tumor grade and *IDH* mutation status using significant metabolites (**A**) High vs low grade (1 vs 0); (**B**) *IDH* positive vs negative (1 vs 0).

Targeted metabolic profiling was performed using LC-QQQ-MS on a total of 87 plasma samples from both the discovery and validation cohorts. The profiling was performed in two phases, discovery (*N* = 42) and validation (*N* = 45). From a targeted 224 metabolites, a total of 157 metabolites were detected in both discovery and validation cohorts. Following log transformation and imputation with minimum observed values for each metabolite, we first attempted to identify metabolites that differed significantly between high- and low-grade gliomas. In the discovery cohort, 8 plasma metabolites differed significantly between high- and low-grade gliomas. They were listed in Table 2. Among them, 5 plasma metabolites were increased in high-grade gliomas; whereas 3 were decreased. The top two significant metabolites were uridine (*P* = 0.004) and ornithine (*P* = 0.016). Compared to low-grade gliomas, levels of plasma uridine were 2.27-fold elevated in high grade gliomas. However, after adjusting multiple comparisons, none of the metabolites was significant (*q* value ≤ 0.05). In the validation cohort, we identified 10 metabolites that differed significantly between high- and low-grade gliomas. They were listed in Table 2. Among them, levels of 6 plasma metabolites were increased in high-grade gliomas and 4 were decreased. The top three significant metabolites elevated in high-grade gliomas were uracil (*P* = 0.007), arginine (*P* = 0.008), and pyroglutamic acid (*P* = 0.016). Levels of plasma uracil were 2.19-fold increased in high-grade gliomas relative to low-grade gliomas. However, after adjusting for multiple comparisons, none of the metabolites was significant. In comparison, of the top 10 significant metabolites identified from the discovery and validation cohorts, 5 significant metabolites (*P* ≤ 0.05) identified in the discovery cohort were also significant in the validation cohort. They were uracil, arginine, lactate, cystamine, and ornithine. Next, we conducted a meta-analysis by combining the samples from discovery and validation cohorts. The heatmap was shown in Supplementary Materials. A total of 29 significant metabolites were identified and top 10 are listed in Table 2. These top 5 metabolites were uridine (*P* = 6.43 × 10^−6^, *q* = 0.001), uracil (*P* = 1.87 × 10^−5^, *q* = 0.001), arginine (*P* = 1.07 × 10^−4^, *q* = 0.005), agmanite (*P* = 1.05 × 10^−4^, *q* = 0.005), and ornithine (*P* = 2.68 × 10^−4^, *q* = 0.005) (Figure 1A). Levels of plasma uridine were 4.86-fold higher in high- than in low-grade gliomas. On the other hand, levels of plasma arginine were 4.02-fold lower in high- than in low-grade gliomas. After adjusting multiple comparisons, there were still 18 significant metabolites left. In further PLS-DA analysis using those 18 significant metabolites, glioma patients could be successfully classified into high- and low-grade groups with 91.1% of accuracy (Figure 2A).

**Table 2:** List of top 10 metabolites in discovery, validation, and combined cohorts*

Discovery (*n* = 42)				Validation (*n* = 47)				Combined meta-analysis (*n* = 89)			
Metabolite	*P* value	Fold change	FDR	Metabolite	*P* value	Fold change	FDR	Metabolites	*P* value	Fold change	FDR
Uridine	0.004	2.27	0.542	Uracil	0.007	2.19	0.301	Uridine	6.43E–06	4.86	0.001
Ornithine	0.016	1.83	0.542	Arginine	0.008	2.12	0.301	Uracil	1.87E–05	4.32	0.001
Cystamine	0.019	1.80	0.542	Pyroglutamic Acid	0.016	1.92	0.301	Arginine	1.07E–04	4.02	0.005
Glucosamine	0.020	1.70	0.542	Lactate	0.028	1.66	0.301	Agmanite	1.05E–04	3.78	0.005
Uracil	0.020	1.73	0.542	Cystamine	0.037	1.52	0.301	Ornithine	2.68E–04	3.46	0.005
Arginine	0.025	1.66	0.542	Ornithine	0.038	1.50	0.301	Biotin	3.54E–04	3.24	0.005
Biotin	0.028	1.53	0.542	Xanthurenate	0.040	1.50	0.305	Lactate	4.27E–04	3.08	0.007
Lactate	0.039	1.42	0.599	Oxalic Acid	0.042	1.42	0.334	Cystamine	9.46E–04	3.09	0.024
				Agmanite	0.046	1.45	0.334	Glucosamine	0.004	2.67	0.024
				Niacinamide	0.047	1.33	0.378	Oxalic Acid	0.002	2.04	0.036

* Metabolites whose fold changes in red reflect an increase accumulation in high grade glioma cases and metabolites whose fold changes in blue reflect a decrease accumulation in high grade glioma cases. The analysis was adjusted by age, gender, ethnicity, KPS, steroid use, seizure medication use, and dyslipidemia diagnosis.

We next investigated whether plasma metabolites could differentiate GBM patients versus malignant glioma patients. Due to the small sample size of GBM, we combined discovery and validation cohorts together (*n* = 18:4 in discovery and 14 in validation). Two metabolites were identified that differed significantly between GBM and malignant glioma patients based on both *P* and *q* values. They were uridine (*P* = 3.76 × 10^−4^, *q* = 0.015) and ornithine (*P* = 9.36 × 10^−4^, *q* = 0.038) (Figure 1B). Then, we explored whether plasma metabolites could differentiate *IDH* mutation status. Altogether, we had 36 patients whose *IDH* mutation status was known. Among them, 12 were negative and 24 were positive. The heatmap was shown in Supplementary Materials. Six metabolites were identified that significantly differed between *IDH* mutation positive and negative tumors based on both *P* and *q* values (Table 3). The top 2 metabolites were N-acetylputrescine (*P* = 9.12 × 10^−4^, *q* = 0.036) and trimethylamine-N-oxide (TMAO) (*P* = 0.006, *q* = 0.043) (Figure 1C). Levels of plasma N-acetylputrescine were 2.96-fold lower in *IDH* mutation positive relative to the negative glioma patients. On the other hand, levels of plasma Methionine were 2.08-fold higher in *IDH* mutation positive than negative glioma patients. In further PLS-DA analysis using those 6 significant metabolites, glioma patients could be successfully classified by *IDH-1* status with 94.4% of accuracy (Figure 2B).

**Table 3:** Significant metabolites differentiating IDH mutation status

Metabolite	*P* value	Fold change	FDR
N-acetylputrescine	9.12E–04	2.96	0.036
trimethylamine-N-oxide (TMAO)	0.006	2.37	0.043
Nicotinate (Niacin)	0.009	2.19	0.043
Arginine	0.009	2.52	0.043
Glucosamine	0.013	1.96	0.047
Methionine	0.016	2.08	0.047

*Metabolites whose fold changes in red reflect an increase accumulation in *IDH* mutation positive cases and metabolites whose fold changes in blue reflect a decrease accumulation in IDH mutation positive cases. The analysis was adjusted by age, gender, ethnicity, KPS, steroid use, seizure medication use, and dyslipidemia diagnosis.

Next, we conducted a pathway analysis which integrates an enrichment analysis and pathway topology analysis. Pathway topology analysis is used to analyze the impact of one metabolite or a group of metabolites in a certain pathway. A total of 18 metabolic pathways were significantly differed between high- and low-grade glioma patients based on a combination of *P* and *q* value (Table 4). Although D-arginine/D-ornithine metabolism and pyrimidine metabolism were the two most significant pathways, the metabolites involved in both pathways have very low impact factors (0 and 0.09, respectively), suggesting they are unlikely to be the metabolic pathways contributing to the difference between high- and low-grade gliomas. On the other hand, arginine/proline metabolism, another significant metabolic pathway (*P* = 0.002 and *q* = 0.010), had high impact factor (0.55). A total of 16 out of 77 metabolites possibly involved in arginine and proline metabolism were analyzed in this study. Among them, 4 differed significantly between high- and low-grade gliomas (*P* ≤ 0.05). They were arginine (*P* = 1.07 × 10^−4^), fumaric acid (*P* = 0.049), agmetine (*P* = 0.007), and ornithine (*P* = 2.68 × 10^−4^). Particularly, arginine and ornithine are key metabolites in the pathway and play critical roles.

**Table 4:** Identification of significant metabolic pathways associated with grade and IDH mutation status

Pathway Name	*P* value	Fold change	FDR	Impact
**High vs low grade**				
D-Arginine and D-ornithine metabolism	1.44E–06	13.45	8.19E–05	0
Pyrimidine metabolism	1.45E–05	11.14	4.14E–04	0.091
Pantothenate and CoA biosynthesis	8.67E–05	9.35	0.002	0.180
Glutathione metabolism	1.29E–04	8.96	0.002	0.002
beta-Alanine metabolism	7.18E–04	7.24	0.008	0.011
Glycolysis or Gluconeogenesis	9.34E–04	6.98	0.009	0.199
Biotin metabolism	0.001	6.73	0.01	0.203
Tryptophan metabolism	0.001	6.5	0.01	0.102
Arginine and proline metabolism	0.002	6.47	0.01	0.553
Amino sugar and nucleotide sugar metabolism	0.002	6.37	0.01	0.091
Pyruvate metabolism	0.003	5.67	0.018	0.32
Propanoate metabolism	0.007	4.93	0.034	0.002
Phenylalanine metabolism	0.011	4.54	0.047	0.198
Glyoxylate and dicarboxylate metabolism	0.012	4.46	0.048	0.162
Tyrosine metabolism	0.013	4.36	0.049	0.162
Taurine and hypotaurine metabolism	0.014	4.28	0.049	0.353
Nicotinate and nicotinamide metabolism	0.015	4.20	0.049	0.135
Methane metabolism	0.016	4.16	0.049	0.018
**IDH mutation status (positive vs negative)**				
Tryptophan metabolism	5.12E–04	7.58	0.019	0.102
D-Arginine and D-ornithine metabolism	6.70E–04	7.31	0.019	0
Arginine and proline metabolism	0.002	6.07	0.043	0.541

**Figure 3:**
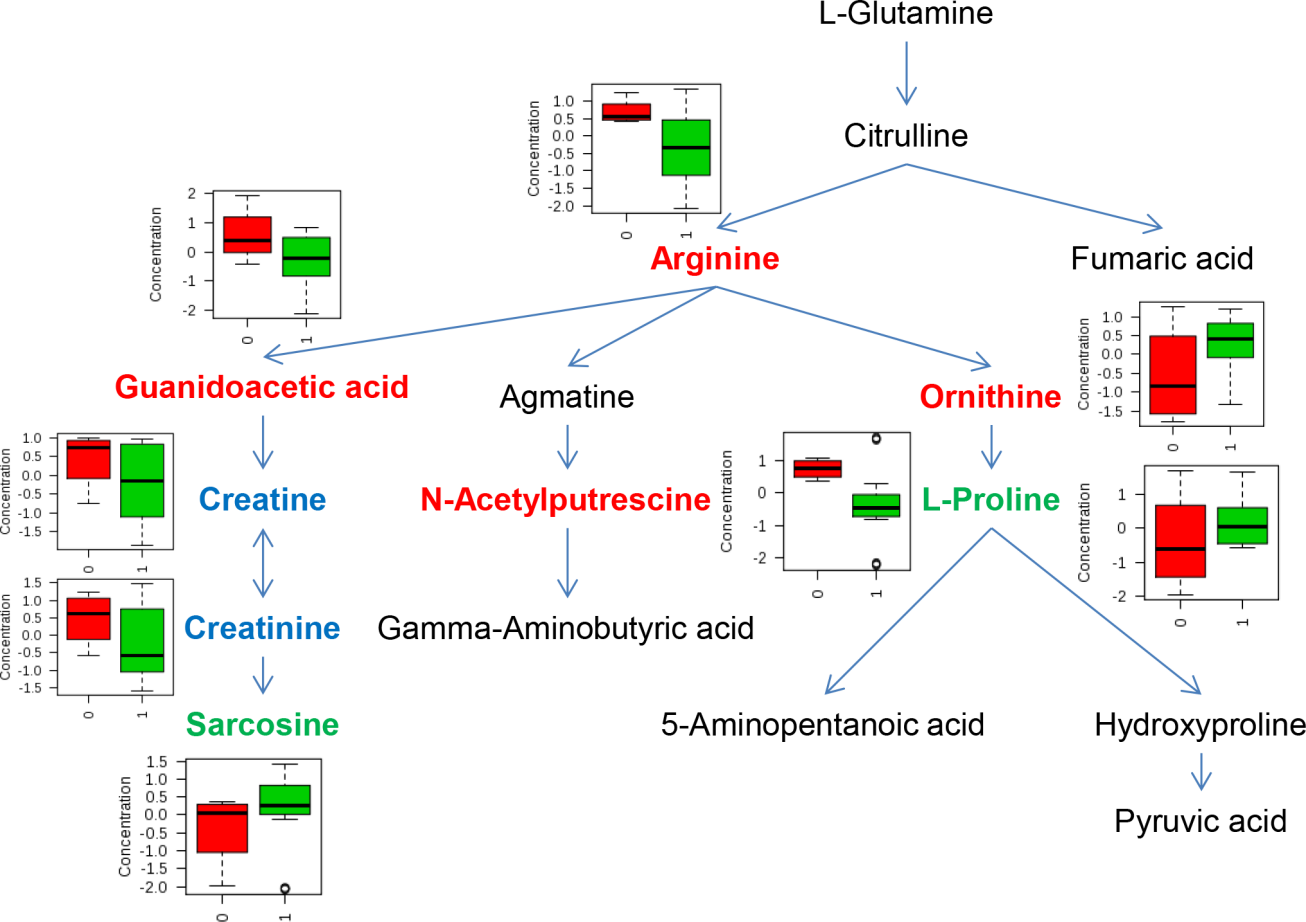
Metabolites involved in arginine and proline pathway that significantly differed by *IDH* mutation status Metabolites in red reflect *P* value ≤ 0.05, metabolites in blue reflect *P* value between 0.05 to 0.10, and metabolites in green reflect *P* value between 0.10 to 0.20. In box-plot, red indicates *IDH* mutation negative and green indicates *IDH* mutation positive.

Using a similar approach, we also studied whether metabolic pathways could differentiate GBM from malignant glioma patients, and *IDH* mutation status. For GBM differentiation, we didn't observe any significant metabolic pathways associated with GBM based on *P* and *q* value. However, we did identify metabolic pathways that significantly differed by *IDH* mutation status (Table 4) such as, tryptophan metabolism, D-arginine and D-ornithine metabolism, and arginine and proline metabolism pathways. When further examined for impact factor, only the arginine and proline metabolism pathway had a high impact factor (0.541). As shown in Figure 3, in arginine and proline metabolism pathway, 4 out of 16 analyzed metabolites had *P* value less than 0.05 (labeled in red). Two metabolites had *P* value ranging from 0.05 to 0.10 (labeled in green), and another 2 had *P* value ranging from 0.10 to 0.20 (labeled in blue). Notably, levels of intermediate metabolites in the creatine pathway such as guanidoacetic acid, creatine, and reatinine were all significantly lower in *IDH* positive than negative patients. At the same time, levels of sarcosine, the downstream metabolite in the pathway, were higher in *IDH* positive than negative patients.

## DISCUSSION

In a recent publication by Eckel-Passow et al., gliomas could be classified into five principal groups on the basis of three tumor markers, including chromosome 1p/19q co-deletion, *IDH* mutation, and *TERT* promoter mutations in tumors [17]. In another study, low-grade gliomas could be further classified into three molecular groups based on *IDH*, *1p/19q*, and *TP53* status [4]. Those studies show the promise of utilizing molecular markers for glioma classification. However, none of the existing studies have looked at the contribution from metabolic homeostasis and how individual difference in metabolic profiles in circulation may correlate with glioma phenotypes. In current study, we used plasma metabolomics profiling to assess the differences between high- and low-grade, GBM and malignant glioma, and *IDH* positive and negative gliomas. Compared to tumor tissues, plasma is a metabolite-rich matrix, readily available via minimal invasive samples techniques, and thereby, is highly amenable to global metabolic profiling studies in gliomas.

In comparison of glioma grade, we identified a total of 29 metabolites in plasma samples that could significantly differentiate high- from low-grade glioma patients. Our results suggest significant changes in pathways related to nucleotides (e.g. pyramidine), amino acids (e.g. arginine, glutathione, alanine, tryptophan), and carbohydrate (e.g. glycolysis and pyruvate) metabolism. This is consistent with the profile of tumor metabolism, which needs to acquire key nutrients from circulation for rapid ATP generation, increased biosynthesis of macromolecules, and maintenance of appropriate cellular redox status [18]. One of the most consistent metabolites observed in this study is arginine, whose plasma levels were significantly lower in high-grade than in low-grade glioma patients in discovery, validation, and combined cohorts. Arginine is a semi-essential amino acid in humans. It has been established that an increased dependence on exogenous arginine is typical for many malignant tumor cells both *in vitro* and *in vivo* [19–20]. Also, a growing number of tumors are being identified as deficient in the arginine anabolic enzyme argininosuccinate synthetase (ASS) and, thus auxotrophic for arginine [21, 22]. Pavlyk et al. showed that arginine deprivation could affect glioblastoma cell adhesion, invasiveness and actin cytoskeleton organization [23]. Thus, the observed difference in plasma arginine levels between high- and low-grade glioma patients could be due to the difference in arginine dependence. Another consistent metabolite is lactate, whose plasma levels were significantly higher in high-grade cases relative to low-grade cases. This is expected since lactate is one of the main metabolic “waste” or by-products during tumor metabolism. Lactate can actually be transported back to the liver to produce glucose (known as the Cori cycle), which imposes an energy burden on whole-body metabolism [24]. Increased flux through this pathway has been postulated to be one of the drivers of energy dissipation in cachexia, which is a contributing factor in one third of all cancer deaths.

*IDH* mutation is not restricted to a specific histopathological type of glioma but instead was associated with a distinctive tumor-cell metabolism [25]. However, how metabolomics profiles in plasma may be differentiated by *IDH* mutation status is unknown. In the current study, we identified 6 plasma metabolites that differed significantly by *IDH* mutation status. The most significant metabolite is N-acetylputrescine. N-Acetylputrescine is the most abundant of all polyamines both in normal individuals and in patients with leukemia [26]. Currently, the role of plasma N-acetylputrescine may play in glioma tumorigenesis is unclear. In the pathway analysis, we found tryptophan metabolism, D-arginine and D-ornithine metabolism, and arginine and proline metabolism were significantly different based on *IDH* mutation status. Within the arginine and proline metabolism pathway, we found levels of intermediate metabolites in the creatine pathway, from guanidoacetic acid, creatine, and creatinine, were all significantly lower in *IDH* mutation positive than in negative patients. However, levels of sarcosine, the downstream metabolite in the pathway, were higher in *IDH* mutation positive than in negative patients. Creatine is usually detected in magnetic resonance imaging research of brain tumors. The creatine levels reflect energy buffering and transport. Our results may suggest an increased activity of creatine pathway in *IDH* mutation positive patients, which is in line with the findings that creatine tended to be low in the high-grade tumors [27]. Among Grade II to IV glioma cases, *IDH* positive status is associated with improved survival [17]. Thus, although survival data are not available in this study yet because of short follow-up time, the plasma metabolites that significantly differed by *IDH* mutation status may potentially have prognostic relevance. Clearly, further investigation of plasma metabolites and survival in glioma patients will be our next step.

Mutated IDH protein leads to the generation of excessive amount of the metabolite 2-hydrocyglutarate (2-HG) in glioma tumor cells [28]. Since 2-HG is a small molecule it seems possible that it could reach the systemic circulation and that elevated 2-HG plasma levels may help to identify patients harboring *IDH* mutated gliomas. In the current study, 2-HG metabolite was detectable. However, no significant association was observed between plasma 2-HG levels and *IDH* mutation status (*P* = 0.458) or tumor grade (*P* = 0.743). In fact, our observation is consistent with the reported from Capper et al. [29]. In their study of 16 glioma patients, no correlation was observed between serum 2-HG levels and IDH1/2 status or tumor size. Thus, 2-HG in circulation may not be a useful marker for glioma molecular classification.

One limitation for circulation metabolomics analysis is the vagueness of the source of the metabolites. In the case of gliomas, tumor cells, tumor infiltrating immune cells and different types of stroma cells all produce metabolites and some of those metabolites could be released and thereby enter the circulation. Currently, there are active studies underway performing metabolic profiling in various types of cancer related cells, including cancer stem cells [30], tumor infiltrating immune cells [31], and other stroma [32–34], in attempt to elucidate their metabolic profiles to dissect the sources of the circulating metabolic factors. The knowledge gained from those studies can help us better interpret the results from this study and provide guidance for circulating biomarker discovery.

In summary, our study showed distinct signatures of plasma metabolite levels by glioma grade (high vs low) and *IDH* mutation status. Additional weaknesses include small sample size, retrospective study design, incomplete exposure information, and the lack of matched tumor/adjacent normal tissues. Nevertheless, our study provides the first evidence to support the role of circulating metabolites in glioma phenotypes. Such knowledge, if confirmed, could help stratify glioma patients and enable personalize medicine.

## MATERIALS AND METHODS

### Study subjects

Case patients were recruited from The University of Texas M. D. Anderson Cancer Center (Houston, TX). All patients with either newly diagnosed or previously treated, histopathologically confirmed glioma who were registered at the Cancer Center between April 2014 and July 2015 were eligible for our study. The exclusion criteria for case patients were currently undergoing chemotherapy or radiation therapy, prior cancer (except for non-melanoma skin cancer), and any blood transfusion in the 6 months prior to recruitment. Written informed consent was obtained from each study subject. A total of 87 glioma patients were included in the study. We divided the study subjects into two cohorts, discovery and validation. The discovery cohort included 42 glioma patients, including 25 low-grade glioma (Grade I: *n* = 5; Grade II: *n* = 20) and 17 high-grade glioma patients (Grade III: *n* = 13; Grade IV GBM: *n* = 4). The validation cohort had 45 glioma patients, including 17 low-grade glioma (Grade I: *n* = 4; Grade II: *n* = 13) and 28 high-grade glioma patients (Grade III: *n* = 14; Grade IV GBM: *n* = 14). The demographic and clinical data for the cases were obtained from medical record review. The study protocol was approved by the Institutional Review Board of The University of Texas M. D. Anderson Cancer Center.

### Metabolic profiling

Metabolomics profiles were obtained using Liquid chromatography triple quadrupole mass spectrometry (LC-QQQ-MS) at Northwest Metabolomics Research Center, University of Washington. Briefly, LC-MS/MS was performed using an electrospray ionization source and the multiple-reaction-monitoring mode. A Sciex 5500 QTRAP triple quad MS system equipped with an Agilent 1200 ultra-high-pressure liquid chromatography system was utilized. The MS acquisition for each plasma sample (0.050 mL each) will target a list of 224 metabolites. In addition, 24 isotope-labeled standards were included in each sample run for quality control and comparisons with other studies or additional samples run at a different time (e.g. discovery vs validation). The average CV of relative intensities for instrument QC samples was 5.3% for the detected metabolites and the average CV of quantified metabolites using isotope-labeled internal standards was 3%, indicating good reproducibility. The targeted 224 metabolites are selected from 25 key metabolic pathways, including alanine, aspartate, and glutamate metabolism; arginine and proline metabolism; butanoate metabolism; the citrate cycle (TCA cycle); cysteine and methionine metabolism; fatty acid metabolism; glutathione metabolism; glycine, serine, and threonine metabolism; glycolysis/gluconeogenesis; histidine metabolism; lysine biosynthesis; lysine degradation; nitrogen metabolism; oxidative phosphorylation; pentose phosphate pathway; phenylalanine metabolism; phenylalanine, tyrosine, and tryptophan biosynthesis; purine metabolism; pyrimidine metabolism; pyruvate metabolism; synthesis and degradation of ketone bodies; tryptophan metabolism; tyrosine metabolism; valine, leucine, and isoleucine biosynthesis; and valine, leucine, and isoleucine degradation. Certain metabolites are involved in multiple metabolic pathways. The raw data are processed using MultiQuant software (AB SCIEX) to integrate chromatographic peaks, and the data are visually inspected to ensure the quality of signal integration. The peak area will be calculated for each individual metabolite.

### Statistical analysis

Any missing values were assumed to be below the limits of detection, and these values were imputed with the compound minimum (minimum value imputation). Statistical analysis of log-transformed data was conducted using MetaboAnalyst 3.0 http://www.metaboanalyst.ca/. Welch *t* tests were conducted to compare data accounted for by estimating false discovery rate (FDR) using *q* values. Logistic regression analysis was applied to assess the relationship between individual metabolite and tumor grade, GBM status, or *IDH* mutation status. Covariates, including age, gender, ethnicity, KPS, steroid use, seizure medication use, and dyslipidemia diagnosis, were adjusted in the analysis. Principal component analysis (PCA) and partial least squares–discriminant analysis (PLS-DA) were carried out using log-transformed data. Metabolic pathway analysis was conducted to identify the enriched metabolite sets and significant metabolic pathways that could differentiate glioma grade (high vs low), GBM versus malignant glioma, and *IDH* mutation status (positive vs negative). The latest version of The Human Metabolome Database (HMDB) 3.0 was used in metabolic pathway analysis [35]. In addition, pathway topology analysis was used to estimate the impact of a certain metabolite or a group of metabolites in a certain metabolic pathway, and relative-betweeness centrality test was used to estimate the impact.

## SUPPLEMENTARY MATERIALS FIGURES


