# Supplementary material for: Metabolomics profiling in plasma samples from glioma patients correlates with tumor phenotypes
Source: Oncotarget. 2016 Mar 7;7(15):20486–95. doi: 10.18632/oncotarget.7974 (PMC4991469; doi:10.18632/oncotarget.7974)
Supplement: Supplementary file 1 [file oncotarget-07-20486-s001.pdf]

Metabolomics profiling in plasma samples from glioma patients correlates with tumor phenotypes

Supplementary Materials

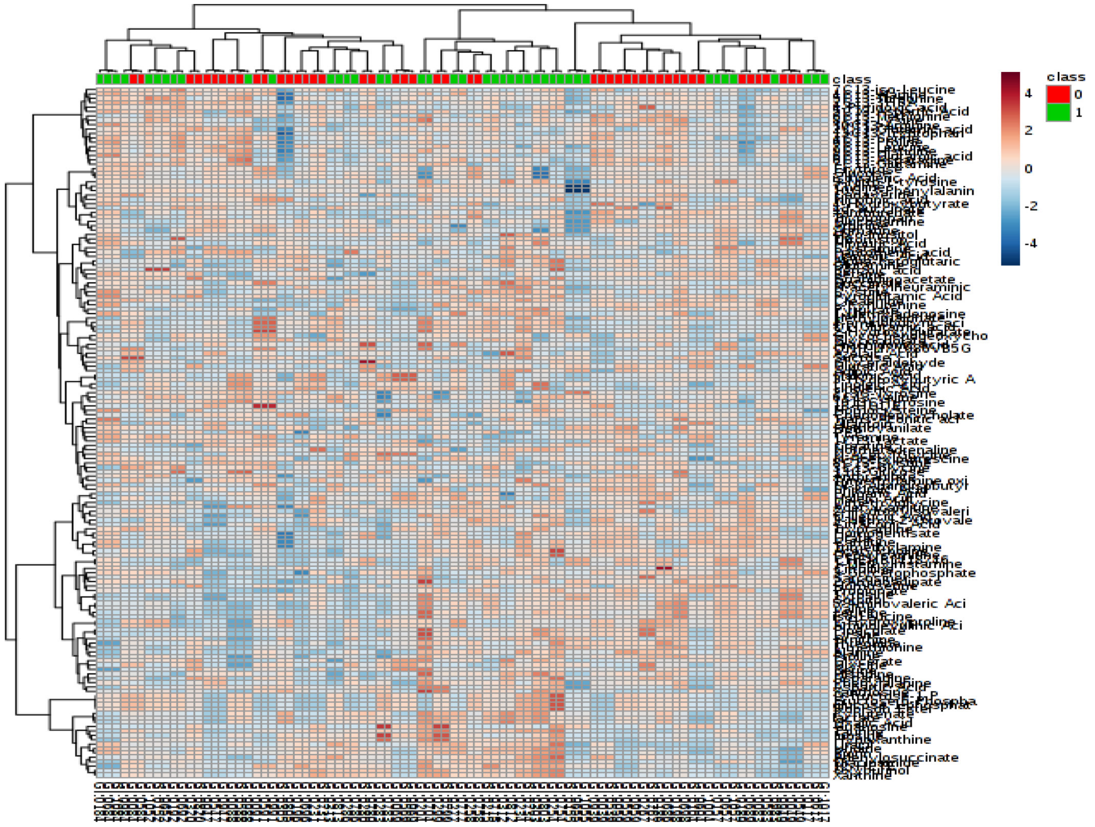

Supplementary Figure S1: Heatmap analysis to differentiate tumor grade (High vs low:1 vs 0).

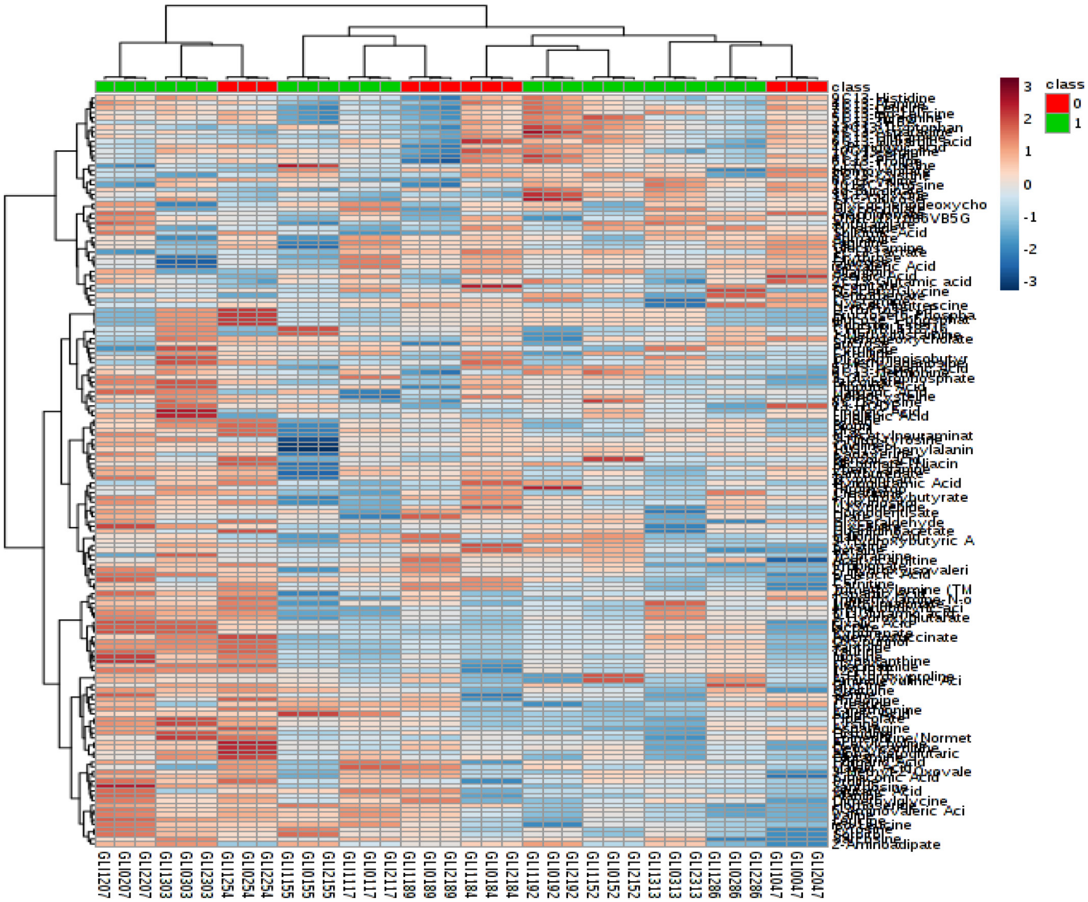

Supplementary Figure S2: Heatmap analysis to differentiate *IDH* mutation status: positive and negative status (1 vs 0).
